# Effect of individualized PEEP titration by ultrasonography on perioperative pulmonary protection and postoperative cognitive function in patients with chronic obstructive pulmonary disease

**DOI:** 10.1186/s12890-023-02471-y

**Published:** 2023-06-28

**Authors:** Lai-feng Luo, Yu-mei Lin, Ying Liu, Xiao-hua Gao, Chui-yu Li, Xiao-qi Zhang, Jian-hua Wu, Zhi-yuan Chen

**Affiliations:** 1 Department of Anesthesiology, the Second Affiliated Hospital of Fujian Medical University, No.950 of Donghai street, Fengze District, Quanzhou, 362000 China; 2 Department of Anesthesiology, The Second Hospital of Sanming, Sanming City, 366000 Fujian Province China

**Keywords:** Chronic obstructive pulmonary disease (COPD), Cognitive function, Individualized PEEP, Interleukin-6 (IL-6), Montreal Cognitive Assessment (MoCA), Pulmonary ultrasound (LUS)

## Abstract

**Objective:**

To evaluate the effect of the individualized positive end-expiratory pressure (PEEP) lung protection ventilation strategy by combining driving pressure (ΔP) and pulmonary ultrasound (LUS)-based titration on lung function and postoperative cognitive function in patients with chronic obstructive pulmonary disease (COPD) during laparoscopic surgery.

**Methods:**

A total of 108 patients with COPD undergoing laparoscopic gastrointestinal surgery under general anesthesia were included in this study. They were randomly divided into three groups (n = 36): traditional volume ventilation group (Group C), fixed PEEP 5 cmH_2_O group (Group P), and ΔP combined with LUS-based PEEP titration in the resuscitation room group (Group T). All three groups were given volume ventilation mode, I:E = 1:2; In group C, VT was 10 mL/kg and PEEP was 0 cmH_2_O; In groups P and T, VT was 6 mL/kg and PEEP was 5 cmH_2_O; After mechanical ventilation for 15 min in Group T, ΔP in combination with LUS was used to titrate PEEP. The oxygenation index (PaO2/FiO2), airway platform pressure (Pplat), dynamic lung compliance (Cdyn), Montreal Cognitive Assessment (MoCA), and venous interleukin-6(IL-6) were recorded at the corresponding time points, and the final PEEP value in Group T was recorded.

**Results:**

The final PEEP value of Group T was (6.4 ± 1.2) cmH_2_O; Compared with groups C and P: PaO_2_/FiO_2_ and Cdyn in Group T were significantly increased (*P* < 0.05) and value of IL-6 was significantly decreased (*P* < 0.05) at the corresponding time points. Compared with group C, the MoCA score on day 7 after surgery in Group T was significantly higher (*P* < 0.05).

**Conclusion:**

Compared with the traditional ventilation strategy, the individualized ΔP combined with LUS-based PEEP titration in patients with COPD during the perioperative period of laparoscopic surgery can play a better role in lung protection and can improve postoperative cognitive function.

## Introduction

In 2020, the World Health Organization (WHO) made the latest prediction on the mortality and cause of death due to chronic obstructive pulmonary disease (COPD). With growing population aging and an increase in the smoking rate, the prevalence of COPD will continue to rise over the next 40 years till 2060. It is predicted that by 2060, the number of patients suffering from COPD and related diseases will be over 5.4 million/year. [1] The mechanism of COPD leading to changes in neurocognitive function has also drawn increasing attention among researchers and academia. Patients with chronic obstructive pulmonary disease (COPD) have been demonstrated to have hypoxemia and hypercapnia for extended periods of time. Chronic hypoxia is associated with neuronal death, degeneration, and necrosis. Hypoxia and the resulting drop in neurotransmitters play a major role in cognitive impairment, and such patients may also experience impaired synthesis of neurotransmitters (acetylcholine). [2–4] According to reports, the incidence rate of postoperative cognitive dysfunction (POCD) one week after surgery is between 9% and 54%. [5] In the long term, POCD also raises postoperative mortality and hospitalization, puts an undue strain on public resources, heightens patient and family anxiety, increases hospitalization costs. [6] Multiple studies have shown that setting an individualized lung protection ventilation strategy during the operation can not only improve lung function of patients, but also improve postoperative cognitive function of patients undergoing surgery and reduce the risk of POCD in elderly patients. [7, 8] The above studies were mainly aimed at lung protection ventilation for healthy lung patients, and there are few studies on individualized lung protection ventilation for patients with COPD during the perioperative period. At present, the lung protective ventilation strategy mainly includes the following elements [9]: Poor postoperative lung compliance can be caused by high tidal volume when low tidal volume ventilation is used (6 ml/kg), however positive end expiratory pressure ventilation (PEEP) can greatly enhance oxygenation, lessen postoperative atelectasis, and increase lung compliance. During laparoscopic procedures, the lung recruitment approach can raise end-tidal lung capacity, boost compliance, and lessen chest wall flexibility. [10] Lung-protective ventilation methods that incorporate individualized PEEP are crucial for halting alveolar collapse and maximizing oxygenation. Common approaches to personalized PEEP titration include the use of electrical impedance tomography, image monitoring, the optimal oxygen method, the ideal lung compliance method, the P-V curve method, the driving pressure method, and the optimal oxygen method. To reduce the perioperative incidence in patients with COPD and reduce the burden on patients and society, we designed an experiment to set up a randomized controlled trial in patients with COPD undergoing laparoscopic gastrointestinal surgery. After evaluating the driving pressure and pulmonary ultrasound, we established the individualized PEEP lung protection and ventilation strategy, and observed the protection of lung function in patients undergoing laparoscopic gastrointestinal surgery and the impact on postoperative cognitive function based on lung protection.

## Methods

### Patients

This clinical experimental study was approved by the Medical Ethics Committee of the Second Affiliated Hospital of Fujian Medical University [(2021) LSZ (No.424)], and informed consent of the patients and their family members were obtained. A total of 108 patients undergoing laparoscopic gastrointestinal surgery combined with COPD under elective general anesthesia were enrolled, with ASA grades I–III, BMI < 30 kg/m^2^, no age or gender limitation, and a certain literacy level. All patients were eligible for FEV1/FVC < 70% following bronchodilator use, to establish a diagnosis of COPD.

#### Anesthesia protocol

Patients in all three groups were given mask inhalation of 100% oxygen for 5 min prior to induction, followed by intravenous injection of propofol 1 mg/kg, sufentanil 0.4 µg/kg, etomidate 0.2 mg/kg, and rocuronium 0.5–0.6 mg/kg; tracheal intubation was performed following mask ventilation for 3–4 min. An anesthesia machine (Drager Tiro, Germany) was connected for the volume-controlled ventilation mode. Intraoperative anesthesia maintenance: Each of the three groups received continuous intravenous and inhaled anesthetic during the procedure. Sevoflurane was administered at 1%, propofol at 3–8 mg/kg, remifentanil at 0.1–0.3 µg/kg min, and atracurium at 0.3–0.6 µg/kg min by inhalation. Vasoactive agents were administered during the procedure to maintain mean arterial pressure within 20% of baseline and BIS values between 40 and 60. Patients were extubated and given mask oxygen in the PACU following surgery; patients with a Steward score > 6 were sent back to the ward 30 min later. The unified intravenous analgesia mode was adopted for all patients.

#### Mechanical ventilation protocol

The patients were divided into three groups according to the random number table, with 36 patients in each group. The traditional volume control ventilation group (Group C): VT = 10*PBW mL/kg, fixed PEEP 0 cm H_2_O group [11]; fixed PEEP 5 cmH_2_O group (Group P): VT = 6*PBW ml/kg, PEEP = 5 cmH_2_O; and driving pressure combined with lung ultrasound titration of PEEP group; (Group T): VT = 6*PBW ml/kg + individualized PEEP. Predicted body weight (PBW) calculated by: Male, PBW = 50 + 0.91* (height-152.4); Female, PBW = 45.5 + 0.91* (height-152.4). Patients were given mask-administered oxygen, subjected to non-invasive monitoring of blood pressure, heart rate, ECG, and pulse oxygen saturation, and had venous access established after they arrived in the operating room. To monitor invasive blood pressure (IBP), a radial artery puncture was conducted while the patient was under local anesthesia with 0.5% lidocaine (IBP). The eight bilateral subareas of the lungs were also examined with ultrasound and scored.

In all three groups, tidal volumes were determined by PBW, and ventilation was performed in a volume-controlled manner. The PetCO_2_ of patients in all three groups was maintained at 35–45 mmHg by regulating respiratory frequency. Group C was given traditional volume ventilation—VT = 10*PBW ml/kg, I:E = 1:2, PEEP value was 0. Group P was given a fixed PEEP value + lung recruitment lung protection ventilation—VT = 6*PBW ml/kg, I:E = 1:2, PEEP = 5 cmH_2_O. Group T was given individualized PEEP value + lung recruitment lung protection ventilation 15 min prior to mechanical ventilation—VT = 6*PBW ml/kg, I:E = 1:2, PEEP = 5 cmH_2_O, and PEEP was set by driving pressure after 15 min of ventilation, and evaluated according to preoperative and postoperative pulmonary ultrasound score.

The specific method is as follows: Basic values were recorded prior to inducing anesthesia, and lung ultrasonography scores were recorded for all four chest quadrants while the patient was in the supine position. Manual lung recruitment was performed after 15 min of mechanical ventilation, and the following manual lung recruitment mode was used: The anesthesia machine’s pressure-limiting valve was adjusted to 30 cmH2O, and the airbag was slowly inflated and kept inflated for 10 s. After that, a pulmonary ultrasonography score was performed after titrating the individual PEEP by driving pressure to find the PEEP value that corresponded to the lowest Pplat-PEEP value. If it was found that the lung ultrasound score was greater than the basic score, manual lung recruitment was performed until the lung ultrasound score in this area was lower, and then the PEEP value was increased by 1–2 cmH_2_O based on the PEEP value corresponding to the minimum value of Pplat-PEEP for ventilation. If there was no change in the four-quadrant score of the chest compared with that prior to induction, the PEEP value was used as the final value for ventilation, and the PEEP value was used for ventilation until the end of the operation if it did not affect the operation.

#### Pulmonary ultrasound scoring method

**Fig. 1:**
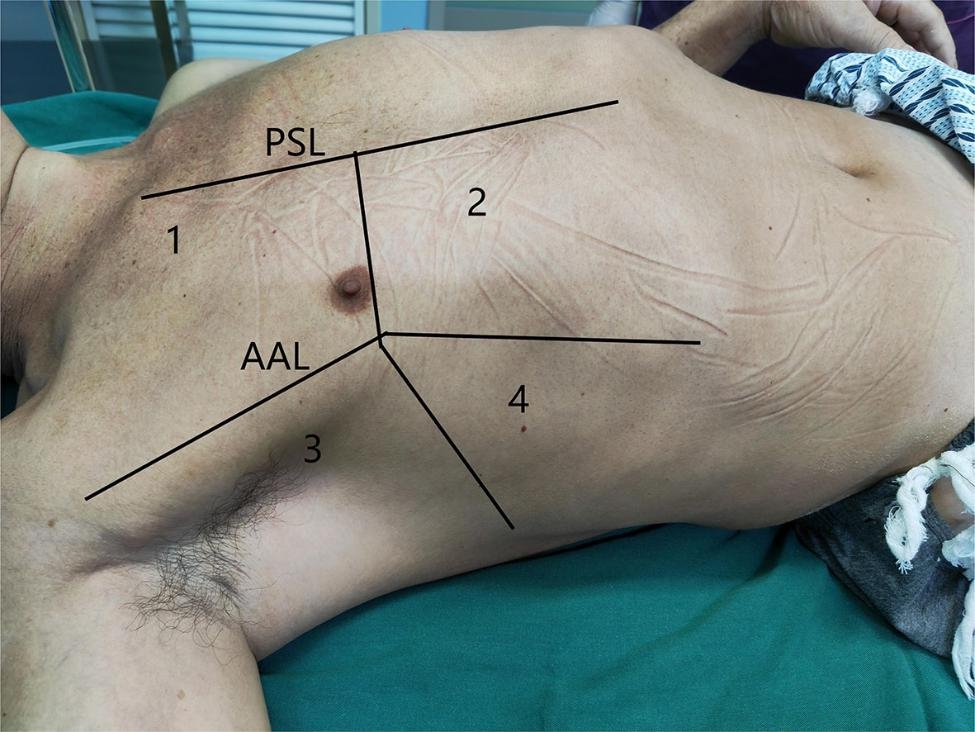
Eight-region lung subarea. Note: Parasternal line (PSL); anterior axillary line (AAL); ultrasound examination of the lungs divided into four quadrants: **1**. Upper anterior lung area; **2**: Anterior inferior lung area; **3**. Superior lung area; **4**. Inferior lung area

**Fig. 2:**
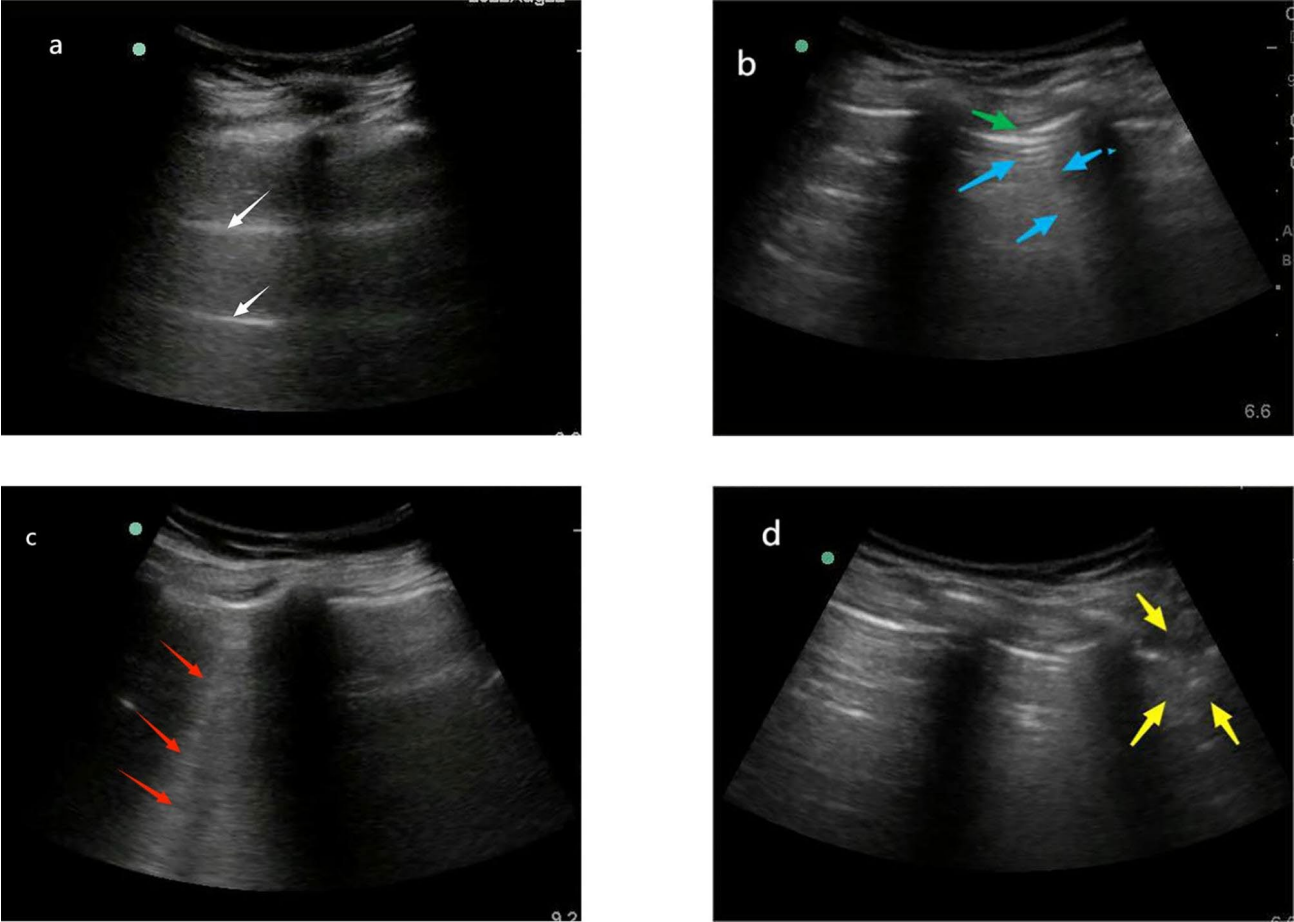
Lung ultrasound images with different scores. Note: The white arrow is the A line; the blue arrow is the B line; the red arrow is the gathering B line; yellow arrows are subpleural nodules; the green arrow is the discontinuous pleura. The above figure shows the ultrasound images of the patients with different scores. Figure **A**: normal pleura and A line, rated as 0; Figure **B**: Discontinuous pleura and multiple B lines, rated as 1 point; Figure **C**: Several gathered B lines, rated as 2 points; Figure **D**: Subpleural consolidation with diameter > 2 cm, rated as 3 points

While the patient was lying in the supine position, the parasternal line and anterior axillary line were used to separate the chest into front and side sections, and the connecting line of the bilateral thoracic nipples was used to split each breast into four quadrants (Fig. 1). The pulmonary ultrasound scoring was conducted according to the experimental method steps described in Monastesse et al. [12] (Fig. 2 shows the images of different ultrasound scoring values for the patient in the experiment). Ultrasonic scoring criteria: Each of the eight quadrants was given a score between 0 and 3 based on the results of the ultrasonic examination, and the total was then calculated. A higher score indicated more severe ventilation loss. Calculation of pulmonary ultrasound (LUS) score (0–24): 0 points, 0–2 B-lines; 1 point, ≥ 3 B-lines or 1 or more subpleural small consolidation separated by normal pleural lines; 2 points, coalescent B lines or multiple subpleural small consolidations separated by thickening or irregular pleural line; 3 points, consolidation or small subpleural consolidation with a diameter > 1*2 cm.

#### Outcomes

The results of the Montreal Cognitive Assessment Scale (MoCA) after the operation, were taken as the primary outcome. The time points were set as follows: one day before surgery (T0), before induction (T1), 15 min after intubation (T2), 30 min after pneumoperitoneum (T3), and at the end of pneumoperitoneum (T4), 30 min after entering the resuscitation room (T5), 2 days after surgery (T6), and 7 days after surgery (T7). At T0, T6, and T7, the three groups of patients were assessed using the MoCA. At T0, T5, and T6, the venous blood of the three groups was taken to check interleukin-6 (IL-6) levels. At T1, T3, T4, and T5, the arterial blood gas was detected, and PaO_2_ and oxygenation index (Pao_2_/FiO_2_ and P/F) were recorded. At T2, T3, and T4, the airway platform pressure (Pplat) and dynamic lung compliance (CDYN) were recorded; at T4, the final PEEP level of group T was recorded. Other general data were recorded including sex, age, hypertension, diabetes, BMI, carbon dioxide pneumoperitoneum time, and volume of intake and output. Removal criteria: The surgery was changed to open surgery during the operation, and the patient was transferred to the ICU after the operation.

#### Statistics and sample size calculation

SPSS19.0 software was used to conduct statistical analysis on the collected data. The measurement data are expressed as mean ± standard deviation or quartile (25%, 75%), and the counting data are expressed in the form of examples and percentages. The variance F test or rank sum test was used for comparison between measurement data groups. Repeated measurement ANOVA was used to compare different time points in the group. The chi-squared test was used to compare the counting data groups. In addition, α = 0.05 was used as the test level for comparison between groups.

According to the results of the previous experiment, when MoCA was used as the primary outcome, Group C, Group P, and Group T had MoCA scores of 23.65 ± 2.39, 25.00 ± 2.00, and 25.95 ± 3.07 on the 7th day following surgery, respectively. The PASS 15.0 software was used to conduct a one-way variance test analysis with α = 0.05 and β = 0.2 set, and the power of this study was 80% for the 36 patients in each group.

## Results

There was no statistically significant difference among the three groups in gender, age, hypertension, diabetes, pneumoperitoneum length, BMI, and output (*P* > 0.05). The final PEEP value in the T group was (6.4 ± 1.2) cm H_2_O and showed statistically significant difference when compared with that in the P group (*P* < 0.05) (Table 1).

**Table 1:** General information of the three groups (n = 36)

Observation index	Group C	Group P	Group T	H/F	*P* value
Gender (Male) (%)	19 (52.8%)	21 (58.3%)	21 (58.3%)	0.301	0.860
Age (${\rm{\bar x}}$ ± s)	61.5 ± 9.6	65.3 ± 8.9	63.8 ± 10.6	1.352	0.263
Hypertension (Yes) (%)	7(19.4%)	9(25.0%)	8(22.2%)	0.321	0.852
Diabetes (Yes) (%)	6(16.7%)	5(13.9%)	7(19.4%)	0.400	0.819
Pneumoperitoneum duration (${\rm{\bar x}}$ ± s) (min)	135.8 ± 44.3	122.1 ± 40.3	128.5 ± 40.0	0.938	0.935
BMI(${\rm{\bar x}}$ ± s)	22.2 ± 2.6	21.9 ± 3.0	21.9 ± 2.3	0.165	0.848
Output (${\rm{\bar x}}$ ± s) ml	538.7 ± 267.5	531.9 ± 237.5	549.1 ± 261.2	0.044	0.957
PEEP values (cm H_2_O)	0	5	6.4 ± 1.2	7.059	< 0.001

Output: the total amount of bleeding and urine during the operation

There was no statistically significant difference in Cdyn among the three groups at the T2 time point (*P* > 0.05, F = 1.086), but there were statistically significant differences at T3 and T4 time points (*P* < 0.05, F = 6.196). Compared with groups C and P, Cdyn in Group T increased significantly at T3 and T4 time points, with statistically significant difference (*P* < 0.05). There was no statistically significant difference of Cdyn in Group T at T4 and T2 time points (*P* > 0.05). Compared within the Cdyn group in groups C and P, T4 was significantly lower than T2, and there was a statistically significant difference (*P* < 0.05) (Table 2).

**Table 2:** Comparison of Cdyn (ml/cmH_2_O) in the three groups at each time point (n = 36, ${\rm{\bar x}}$ ± s)

Time	Group C	Group P	Group T	F	*P* value
**T2**	32.58 ± 4.77	33.47 ± 5.73	34.81 ± 8.40	1.086	0.341
**T3**	20.56 ± 2.65	22.50 ± 4.08	25.87 ± 4.20^a^	18,874	< 0.001
**T4**	27.94 ± 3.97^ cd^	28.7 ± 4.90^ cd^	31.50 ± 4.54^bcd^	6.196	0.003

Note: Compared with groups C and P, at T3 and T4, ^a^*P* < 0.05, ^b^*P* < 0.05. In group T: Compared with T2, ^*c*^*P > 0.05*; Compared with T3, ^*d*^*P < 0.05*. In Group P: Compared with T2, ^*c*^*P < 0.05*; Compared with T3, ^*d*^*P < 0.05*. In Group C: Compared with T2, T3, ^*c*^*P < 0.05*, ^*d*^*P < 0.05*

There was no statistically significant difference in P/F among the three groups at the T1 time point (*P* > 0.05, H = 0.059), but there were statistically significant differences at T3, T4, and T5 time points (*P* < 0.05). Compared with groups C and P, P/F in Group T was significantly increased at T3 and T5 time points, and there was a statistically significant difference (*P* < 0.05). Compared with Group C, P/F in groups T and P increased significantly at the T4 time point, with statistically significant difference (*P* < 0.05). There was no statistically significant difference in P/F within Group T at T5, T4, and T1 time points (*P* > 0.05). Compared with T4, T3, and T1 time points, P/F and T5 within groups C and P decreased significantly, with a statistically significant difference (*P* < 0.05). There was no statistically significant difference in P/F within Group P at T4, T3, and T1 time points (*P > 0.05*) (Table 3).

**Table 3:** P/F at each time point in the three groups (n = 36, ${\rm{\bar x}}$ ± s)

Time	Group C	Group P	Group T	F /H	p
T1	423 ± 74	415 ± 58	416 ± 55	0.059	0.971
T3	398 ± 62	418 ± 55	454 ± 51^a^	9.1	< 0.001
T4	377 ± 59^e^	423 ± 40^bd^	445 ± 40^b^	15.9	< 0.001
T5	340 ± 104^c^	67 ± 56c	417 ± 52^ac^	11.6	< 0.001

Note: Compared with groups C and P, at T3 and T5, ^*a*^*P* < 0.05; Compared with Group C, at T4, ^*b*^*P* < 0.05; In Group T: Compared with T1 and T4, ^*c*^*P* > 0.05; In Group P: Compared with T1, T3, and T4, ^*c*^*P* < 0.05, compared with T1 and T3, ^*d*^*P* > 0.05; In Group C: Compared with T1, T3, and T4, ^*c*^*P* < 0.05, compared with T1, ^*e*^*P* > 0.05

There were statistically significant differences in IL-6 values among the three groups at the T6 time point (*P* < 0.05). Compared with groups C and P, the IL-6 value in Group T decreased significantly at T6 time point, and there was a statistically significant difference (*P* < 0.05). Compared with the IL-6 value within Group T, it was significantly lower at T6 than at T5, with a statistically significant difference (*P* < 0.05). The values of IL-6 in groups C and P at the T6 and T5 timepoints were significantly higher than those at T0, and there was a statistically significant difference (*P* < 0.05); compared with T5, T6 was improved, but there was no statistically significant difference between T6 and T5 (*P* > 0.05) (Table 4).

**Table 4:** Comparisons of IL-6 (ng/ml) in the three groups at each time point

Time	Group C, n = 36	Group P, n = 36	Group T, n = 36	Chi-square value	*P* value
T0	(0.003975,0.015200)	(0.003675,0.014175)	(0.002900,0.012500)	1.728	0.422
T5	(0.018950,0.088600)	(0.014350,0.069775)	(0.012925,0.033750)	5.937	0.051
T6	(0.017500,0.070075)^c^	(0.022575,0.061775)^c^	(0.009400,0.019550)^abc^	28.134	< 0.001

Data were expressed as quartile (25%, 75%)

Note: Compared with groups C and P, at T6, ^*a*^*P* < 0.05. In Group T: Compared with T5 and T0, ^*b*^*P* < 0.05; Compared with T5, ^*c*^*P* < 0.05; Group P: Compared with T5, ^*c*^*P* > 0.05; Group C: Compared with T5, ^*c*^*P > 0.05*.

The MoCA scores of the three groups had statistically significant difference at time point T7 (*P* < 0.05). Compared with Group C, the MoCA score of Group T was significantly higher at time point T7, and there was a statistically significant difference (*P* < 0.05). There was no significant difference in MoCA scores in Group T at T1, T6, and T7 time points (*P > 0.05*). The MoCA scores at T7 and T6 time points in groups P and C were significantly lower than those at time point T1, and there were statistically significant differences (*P* < 0.05). Table 5.

**Table 5:** Comparison of MoCA in the three groups at each time point, n = 36, quartile (25%, 75%)

Time	Group C	Group P	Group T	Chi-square value	*P* value
T0	(19.00,27.75)	(18.50,27.00)	(17.00,26.75)	0.807	0.668
T6	(14.50,24.00)	(16.50,23.75)	(14.25,24.00)	0.059	0.971
T7	(12.25,22.00)^cd^	(17.00,23.75)^cd^	(16.00,26.00)^ab^	7.978	0.019

Note: Compared with Group C, at T7 time point, ^*a*^*P < 0.05*. In Group T: Compared with T6 and T0, ^*b*^*P > 0.05*; In Group P: Compared with T6, ^*c*^*P > 0.05*, compared with T0, ^*d*^*P < 0.05*; Group C: Compared with T6, ^*c*^*P > 0.05*, compared with T0, ^*d*^*P < 0.05*

## Discussion

Previous research has shown that applying protective ventilation to the lung during mechanical ventilation after surgery improves oxygenation and lung mechanics, decreases the loss of alveolar switch and lung elastic retraction force, raises lung compliance, and boosts lung function. [11, 13–15] There have been many studies on the setting of PEEP level during surgery, but they have remained controversial. [16, 17] In clinical practice, the driving pressure method (ΔP, calculated as the platform pressure Pplat minus PEEP) has attracted much attention to titrate individualized PEEP; we considered the corresponding PEEP value when the minimum driving pressure was selected. A low ΔP may reduce major postoperative pulmonary complications (PPCS), including acute respiratory distress syndrome (ARDS), pneumonia, pulmonary edema, need for reintubation, pulmonary infection, and lung barotraumas. [18] However, as ΔP is affected by respiratory parameters and lung compliance, it is not known whether ΔP is also suitable for laparoscopic surgery in patients with COPD. In this study, we studied the protection of lung function by using ΔP combined with lung ultrasound score to evaluate individualized PEEP for patients with COPD undergoing laparoscopic abdominal surgery. The lung ultrasound score is less impacted by respiratory parameters and lung compliance on ΔP. Intuitive image guidance of lung ultrasound can ensure double lung ventilation during perioperative mechanical ventilation and prevent the occurrence of atelectasis or insufficient ventilation during perioperative mechanical ventilation. In this study, the final value of (6.4 ± 1.2) cmH_2_O in the individualized PEEP group was statistically different from that in the fixed PEEP 5 cmH_2_O group; and the lung compliance in the individualized PEEP lung protection ventilation group was higher than that in the fixed PEEP value group and the traditional volume ventilation group at 30 min and the end of pneumoperitoneum. This finding implies that PEEP values of 5 cmH2O and below in the fixed-value PEEP group and the traditional volume ventilation group are insufficient to sustain the opening of the alveoli, leading to partial alveolar collapse once again during the procedure. Patients in the individualized PEEP group, however, showed greater individual variation and could more flexibly maintain alveolar openness and lung compliance. At the same time, the lung oxygenation of patients in the individualized PEEP group was significantly higher than that in the fixed-value PEEP group and the traditional lung ventilation group. There was no statistically significant difference in the oxygenation index between resuscitation room + at the end of pneumoperitoneum and before operation in the individualized PEEP group—this may be due to the proper PEEP value being maintained in the individualized PEEP group, which can aid in opening and maintaining alveoli, so that oxygen molecules can better diffuse into the blood; these results are consistent with previous studies. The results of this study show that driving pressure combined with lung ultrasound titration of individualized PEEP can better titrate PEEP for patients with COPD. Combined with lung recruitment, it can keep more alveoli open, improve gas exchange, improve the oxygenation function and lung compliance, prevent postoperative pulmonary complications, and play a protective role in lung function to some extent.

Mechanical ventilation and surgical trauma increase pulmonary vascular permeability, leading to pulmonary edema, pulmonary infection, and systemic inflammatory response syndrome. This is because they stimulate inflammatory cells, activate multiple inflammatory signal transduction pathways, and produce numerous inflammatory cytokines. [19–21] The existing evidence of medium-and high-quality observational studies indicate that POCD is related to the concentration of peripheral inflammatory markers, and some of these markers, such as CRP and IL-6, play a role in POCD. [22–25] In this study, the driving pressure combined with individualized LUS-based PEEP titration could better improve the lung compliance of patients during the operation. Compared with fixed-value PEEP and traditional volume ventilation, it could significantly increase the oxygenation of patients during the perioperative period and has the effect of significantly improving lung function. IL-6 levels of the individualized PEEP group on the 2nd day after operation were significantly lower than those of the fixed-value PEEP group and the traditional volume ventilation group. IL-6 level on the 2nd day after operation was significantly lower than that in the resuscitation room 30 min after operation. IL-6 values in both groups of the fixed-value PEEP group and the traditional volume ventilation group were significantly increased compared with those on the 2nd day after surgery and after 30 min in the resuscitation room, and the improvements on the 2nd day after surgery compared with those in the resuscitation room for 30 min showed no statistically significant difference. At the same time, the MoCA scores of the individualized PEEP group on the 2nd and 7th days after operation were higher than those of the other two groups, and there was statistically significant difference. There was no statistically significant difference between the pre-operation and post-operation MOCA scores in the individualized PEEP group. The MoCA scores on the 2nd and the 7th days after surgery in the fixed-value PEEP group and the traditional volume ventilation group were significantly lower than those before surgery, and there were statistically significant differences. There was no statistically significant difference in MOCA scores on the 2nd and the 7th days after surgery between the fixed PEEP value group and the traditional volume ventilation group. Through the results of this study, it is evident that the driving pressure and LUS-based PEEP titration strategy can reduce the IL-6 level of patients with COPD after laparoscopic surgery and improve the postoperative cognitive function score, and it plays a certain role in improving and preventing the cognitive dysfunction of patients after surgery.

Patients with a body mass index (BMI) > 30 were excluded from this study because of the known association between obesity and decreased residual lung volume and increased lung complications; however, the study did not restrict or compare patients based on their COPD severity or preoperative lung function, which could introduce some population bias. Furthermore, this study may have been biased due to the difficulty in studying intrinsic PEEP and air trapping in patients with COPD undergoing mechanical ventilation due to the unpredictable shifts in patient position and pneumoperitoneal pressure that occur during laparoscopic surgery. The MoCA score at T6 for Group T was lower than the MoCA score at T7 in this study. Changes in cognitive function in patients with COPD after general anesthesia need to be monitored, as demonstrated by declines in MoCA score from baseline (T0) in the three study groups. Patients with COPD who receive individualized PEEP have better cognitive function after surgery, as evidenced by the higher MoCA score at T7 in Group T. In this study, we used a manual recruitment technique, although there is mounting evidence that such an approach negatively affects patient outcomes, suggesting that a mechanical recruitment maneuver be used instead in future research and clinical practice.

There are some limitations to this study: (1) The patients included in this study were patients who underwent laparoscopic gastrointestinal surgery. Due to the different surgical positions, the pressure factors of the position on the chest cavity were not considered. Patients should be recruited for future trials based on their surgical position and procedure type to minimize the impact of the surgical positions on the research results and increase the accuracy of the research. (2) In this study, PEEP titration and lung ultrasonography examination were carried out only once following tracheal intubation to avoid interrupting the surgical procedure. It is possible that the research results do not accurately reflect the actual scenario because individual PEEP titration was not done throughout the surgery, coupled with the shift in body posture and the extension of the operation period. Individualized PEEP titration should, in theory, be performed every 30 min at most. (3) The results of this investigation should be confirmed by future studies with larger samples and more participants, as it is a single-center study with a limited sample size. (4) We were unable to examine the effect of individualized PEEP lung protection ventilation on long-term cognitive function following surgery due to our focus on short-term outcomes within the first seven days after surgery and our failure to assess MoCA scores at later time points.

In conclusion, using the driving pressure approach and pulmonary ultrasound assessment as part of an individualized PEEP titration lung protection ventilation strategy can improve perioperative lung function in patients with COPD and safeguard postoperative cognitive function.

## Data Availability

All data generated or analysed during this study are included in this article. Further enquiries can be directed to the corresponding author.
